# Choline and docosahexaenoic acid during the first 1000 days and children’s health and development in low- and middle-income countries

**DOI:** 10.1093/nutrit/nuab050

**Published:** 2021-08-02

**Authors:** Megan G Bragg, Elizabeth L Prado, Christine P Stewart

**Affiliations:** Institute for Global Nutrition, University of California Davis, Davis, California, United States

**Keywords:** child growth, choline, DHA, neurodevelopment, pregnancy

## Abstract

Choline and DHA are nutrients that, when provided during the first 1000 days from conception to age 2 years, may have beneficial effects on child neurodevelopment as well as related health factors, including birth outcomes and child growth, morbidity, and inflammation. Because these nutrients are found mainly in animal-source foods, they may be lacking in the diets of pregnant and lactating women and young children in low- and middle-income countries, potentially putting children at risk for suboptimal development and health. Prior reviews of these nutrients have mainly focused on studies from high-income countries. Here, a narrative review is presented of studies describing the pre- and postnatal roles of choline, docosahexaenoic acid, and a combination of the 2 nutrients on child neurodevelopment, birth outcomes, growth, morbidity, and inflammation in low- and middle-income countries. More studies are needed to understand the specific, long-term effects of perinatal choline and docosahexaenoic acid intake in various contexts.

## INTRODUCTION

The period from conception through the first 2 years after birth (termed the “first 1000 days”) is a time of rapid neurodevelopment when developmental trajectories are malleable to contextual exposures, with outcomes negatively affected by risk factors and positively affected by interventions.^1^ Nearly 250 million children younger than 5 years in low- and middle-income countries (LMICs) are at risk for not reaching their developmental potential , which can have adverse effects on future schooling, productivity, and health.^2^ Although many environmental conditions influence neurodevelopment, nutrition is a major component.^3^ Nutrition also affects factors such as preterm birth, childhood stunting, morbidity, and inflammation, which are common in LMICs and linked to impaired neurodevelopment.^4^ Choline and docosahexaenoic acid (DHA) are 2 nutrients that may influence child neurodevelopment, as well as birth outcomes, growth, morbidity, and inflammation.

Both choline and DHA can be endogenously produced from precursors; however, it is unlikely that endogenous production is sufficient to meet needs, so recommended intake levels have been established for pregnant and lactating women and young children (Table 1).^5^^,^^6^ Except for DHA requirements for women, these recommendations are based on adequate intake levels and may over- or underestimate needs. Choline recommendations, in particular, are based on few studies and do not consider neurodevelopmental outcomes. Whereas the World Health Organization developed the DHA guidelines with the Food and Agriculture Organization of the United Nations, there are no global guidelines for choline intake.

**Table 1:** Recommended intake levels for choline and docosahexaenoic acid in pregnant or lactating women and young children

	Choline (mg/d)^a^	DHA^b^
Pregnant women	450	200 mg/d
Lactating women	550	200 mg/d
Infants aged 0–6 mo	125	0.10%–0.18% of total energy
Infants aged 7–12 mo	150	10–12 mg/kg
Children aged 1–2 y	200	10–12 mg/kg

*Abbreviation:* DHA,docosahexaenoic acid.

a Adequate intake levels set by the United States Institute of Medicine.^5^

b Average nutrient requirement levels (for women) and adequate intake levels (for infants and children) set by the Food and Agriculture Organization of the United Nations, World Health Organization.^6^

The main food sources of choline and DHA are of animal origin, such as eggs and fish. Because animal source foods may be expensive,^7^ intake of choline and DHA may be limited in many LMICs. Processed foods may also provide choline as lecithin; as processed food consumption increases in LMICs, this may become a significant source. Breast milk is a rich source of choline and DHA for young children, although concentrations of both nutrients vary on the basis of maternal intake.^8^^,^^9^ Foods contain different forms of choline (free choline, phosphocholine, glycerophosphocholine, phosphatidylcholine, and sphingomyelin); each form should be included in estimates of total choline intake.^10^

A lack of representative food composition estimates in national food databases limits assessment of dietary intake; however, it seems intake often falls below recommended levels in LMICs. For example, in a review that reported choline intake in 15 countries, intake among women was lowest in Mexico (263 mg/d) and highest in Sweden (374 mg/d).^10^ Another study in The Gambia reported even lower intakes of choline (155.2 mg/d) among 62 nonpregnant women of reproductive age.^11^ Intake of DHA below recommendations is also common in LMICs. According to food balance sheets from 175 countries, per capita availability of DHA among low-income countries was 96 mg/day compared with 184–473 mg/day across high-income countries.^12^ Using similar data plus breastfeeding rates, the median DHA intake among children aged 6–36 months in LMICs was estimated to be 48.7 mg/day, well below recommendations.^13^ These nutritional inadequacies may put children at risk for suboptimal development and may be exacerbated by other common health factors in LMICS. These include inadequate intake of other nutrients, such as iron, zinc, and vitamin B_12_, required for endogenous production of DHA and choline,^6^^,^^14^ as well as conditions like gestational diabetes mellitus and an altered composition of the gut microbiota, which may affect DHA and choline availability, respectively.^15^^,^^16^

The relationship between poor intake and outcomes is clouded by limitations in assessing choline and DHA status. Plasma choline is poorly correlated with intake across a range of intake levels,^11^^,^^17^ and may be affected by plasma volume expansion in pregnancy. Lipid-soluble forms of choline (such as phosphatidylcholine) are influenced by fat metabolism and transport in lipoproteins. For DHA, red blood cell (RBC) concentration is a better marker of habitual exposure than is plasma concentration, although sample collection and storage are more difficult.^18^

Few reviews have examined choline and DHA together. Choline and DHA are present in many of the same food sources and are hypothesized to have similar effects on neurodevelopment, especially memory and learning. Their metabolism is also linked—phosphatidylcholine molecules can incorporate DHA, as we describe later in this article—and there is evidence that these nutrients work synergistically to promote neurodevelopment.^19^ Several reviews have focused on either choline or DHA, presenting evidence from predominantly high-income countries.^20–23^ This review presents the evidence relating choline, DHA, and a combination of the 2 nutrients during the first 1000 days of life to children’s neurodevelopment, birth outcomes, growth, morbidity, and inflammation in the context of LMICs.

## METHODS

Literature searches were performed in PubMed using the terms: choline, DHA, docosahexaenoic acid, fish, egg; pregnancy, lactation, complementary feeding, formula, infant; child development, neurodevelopment, cognition, memory, vision, visual; child growth, height, weight, head circumference; preterm, birth; morbidity; diarrhea; enteropathy; inflammation; as well as a list of LMICs based on World Bank income categories in 2019–2020. Abstracts and titles were screened for inclusion. Reference lists were scanned for eligible studies.

Selected papers included observational studies or randomized controlled trials (RCTs) in LMICs. Relevant animal studies were included in discussions of mechanisms; however, only human trials were included in discussions of the evidence in LMICs. All types of interventions were eligible, including supplements, foods, or dietary advice to consume foods rich in choline and/or DHA, and studies could include co-interventions, such as arachidonic acid (ARA) or eicosapentaenoic acid (EPA). Studies published in English by August 11, 2020, were eligible.

The outcomes assessed were neurodevelopment (behavioral and physiological measures), physical growth (height, weight, head circumference, measures of adiposity), birth outcomes (preterm birth/gestational age, birth length, birth weight), morbidity (illnesses such as diarrhea, environmental enteropathy), and biochemical markers of inflammation. Visual development was included with neurodevelopmental outcomes, when available. Morbidity and inflammation data are presented together because of the limited number of studies identified. Outcomes could be measured at any age; however, the initial exposure assessment must have been during pregnancy or the first 2 postnatal years. Articles were organized by the nutrient of interest (choline, DHA, or both) and the timing of exposure (prenatal, postnatal, or across both periods).

### Choline

#### Proposed mechanisms

Choline is an essential micronutrient that is important for early neurodevelopment. In rodent studies, clear improvements were observed in lifelong memory when choline was supplemented during specific pre- and postnatal periods,^24–26^ in part by altering rates of mitosis and apoptosis of neural progenitor cells in the hippocampus and the cerebral cortex.^27^^,^^28^ These effects are epigenetically mediated through conversion of choline to the methyl donor betaine.^29^ Betaine donates a methyl group to homocysteine to form methionine and eventually *S*-adenosyl methionine. These epigenetic changes may affect neurodevelopment in indirect ways, as well. For example, high maternal intake of choline decreases placental expression of cortisol-stimulating genes, with potential effects on learning and memory.^30^ Choline may also affect development in its role as a precursor of phosphatidylcholine and acetylcholine. Phosphatidylcholine is a major component of cell membranes and a precursor of sphingomyelin, required for myelination of neurons, and the cell-signaling molecule diacylglycerol. Acetylcholine is a neurotransmitter involved in the encoding of new memories in the hippocampus; it is also a neuromodulator that influences neurogenesis and synapse formation.^31^

Compared with neurodevelopment, there is less mechanistic evidence for choline’s role in birth outcomes, child growth, morbidity, and inflammation. In rodent models, prenatal choline supplementation modulates nutrient transport across the placenta, increasing choline availability and altering glucose and amino acid metabolism.^32^ As a methyl donor, choline may reduce homocysteine levels, which are associated with adverse pregnancy outcomes,^33^ and increase vitamin B_12_ availability in pregnant women.^34^ Perinatal choline may also influence bone growth and body size. Rodent knockout models without the choline kinase enzyme (which converts choline to phosphocholine) have altered bone formation,^35^^,^^36^ and phosphatidylcholine is required for the production of new cell membranes. Related to morbidity and inflammation, choline supplementation in rodents reduced markers of inflammation after lipopolysaccharide administration during pregnancy.^37^ Different forms of prenatal choline (eg, free choline or phosphatidylcholine) also may affect development of the offspring immune system.^38^ Choline’s roles outside of neurodevelopment are active areas of research.

#### Choline during pregnancy

##### Designs of reviewed studies

Two RCTs in LMICs have been conducted to study prenatal choline supplementation in human populations. In South Africa, heavy alcohol consumers were randomly assigned to choline (2 g/d) or placebo from mid-pregnancy until delivery (*n* = 69).^39^ Although baseline plasma choline concentration was not reported, mean choline intake at baseline was below guidelines for pregnant women (∼ 370 mg/d in both groups). In a trial in Ukraine, researchers also examined the effect of choline among women who consumed alcohol during pregnancy (*n* = 163); however, this trial enrolled abstaining pregnant women, as well (*n* = 204).^40^^,^^41^ Women were randomly assigned to a daily multiple micronutrient supplement (MMS) with 750 mg of choline, MMS alone, or standard of care (ie, no provision of supplements) from the first prenatal visit until delivery. The subgroups that received choline were small (*n* = 19 alcohol consumers and *n* = 18 alcohol abstainers). Baseline plasma choline levels were similar across groups (∼ 15 µmol/L). The primary outcome of both trials was neurodevelopment during the first year of life. One observational study in China reported associations of maternal plasma choline with birth outcomes.^42^ We found no studies in LMICs that reported on prenatal choline and infant morbidity or inflammation.

##### Neurodevelopment

In the South African trial, infants in the choline group had improved eye-blink conditioning, an early marker of learning and memory, than did control infants at 6.5 months; however, this was only significant after removing 4 infants in the choline group whose mothers were considered to have poor adherence. The choline group also had significantly higher mean novelty preference scores on the Fagan Test of Infant Intelligence compared with control children at 12 months (64.5% vs 59.1%; *P* < 0.05), demonstrating improved visual recognition memory. There were no effects on information processing speed at 6.5 or 12 months.^39^ In the Ukrainian trial, addition of choline to MMS did not significantly affect Bayley Scales of Infant Development (BSID) II Psychomotor Development Index or Mental Development Index scores at 4–11 months.^41^ However, infants in the choline group demonstrated improved encoding and memory of visual stimuli, as measured by larger and faster changes in heart rate during habituation and dishabituation tasks at 4–11 months.^40^

Together, the findings from these 2 studies suggest neurodevelopmental benefits in the first year from prenatal supplementation of choline doses from 750 mg–2 g/d, although this may be primarily generalizable to women who consume alcohol during pregnancy. More studies are required with abstaining women in LMICs and with prolonged follow-up to assess the long-term effects of prenatal choline supplementation. Detecting effects of choline may depend on the neurodevelopmental assessment methods used. Assessments of attention and memory based on eye-blink, eye movements, and heart rate may be more sensitive than assessments based on acquisition of developmental milestones, such as the BSID.

##### Pregnancy outcomes

In the South African trial, there was no difference between groups in mean gestational age (choline, 38.8 weeks vs control, 38.9 weeks) or incidence of low birth weight (LBW) (25.0% vs 32.3%), although mean birth length was nonsignificantly lower in the choline group (47.2 cm [SD, 3.3] vs 48.9 cm [SD, 3.7]; *P* < 0.1).^39^ In Ukraine, birth outcomes were compared by maternal supplementation (MMS vs standard of care; MMS with choline vs MMS alone) and alcohol consumption. Children whose mothers received the MMS with or without choline had significantly higher birth weight compared with the standard-of-care control group, a pattern that was evident among those born to women who consumed alcohol during pregnancy and those born to women who abstained. However, when contrasting the group who received MMS plus choline with MMS alone, birth weight was significantly lower (−126 g among mothers who consumed alcohol, and −171 g among abstaining mothers; *P* = 0.048).^40^ In an observational study of 115 pregnant women in China, maternal plasma choline was not associated with birth outcomes, although the choline metabolite betaine was inversely associated with birth weight.^42^

The scant information available suggests additional prenatal choline may be related to smaller birth size; however, this reflects the findings of only 2 small trials that enrolled women who consumed alcohol during pregnancy, neither of which were designed to investigate birth outcomes. Future studies should explore the link between prenatal choline supplementation and birth size in LMICs.

##### Child growth

In the South African trial, the control group decreased in weight, length, and head circumference *z* scores over the first year; in contrast, the choline group experienced catch-up growth in weight percentile and head circumference percentile from birth to 12 months.^39^ No studies reported on growth after prenatal choline supplementation among women who did not consume alcohol.

#### Choline from birth to 2 years

##### Designs of reviewed studies

No trials of early postnatal (0–2 years) choline supplementation in LMICs were identified. Three observational studies reported on the association of choline and growth within this life stage in Malawi,^43^ Brazil,^44^ and Bangladesh.^45^ No studies reported on early postnatal choline supplementation and child neurodevelopment, morbidity, or inflammation in LMICs.

##### Child growth

In a cross-sectional study of 325 Malawian children aged 12–59 months, researchers observed a difference of 0.41 cm in height per 1 SD difference in serum choline (*P* < 0.0001), with a larger magnitude in boys (0.60 cm) than in girls (0.19 cm).^43^ Ratios of betaine to choline and trimethylamine *N*-oxide to choline, representing choline conversion to metabolites, were also investigated; both ratios were negatively associated with children’s height-for-age *z* scores (HAZ).

In Brazil, urinary metabolites were measured among 326 children age 6–24 months with weight-for-age *z* scores (WAZ) of less than −2 or greater than −1. Children with a WAZ less than −2 had lower concentrations of urinary choline metabolites, signifying changes in choline metabolism among underweight children.^44^ In a metabolomics study of 130 Bangladeshi children, sphingomyelins and phosphatidylcholine species were positively associated with change in HAZ from 9 months to 4 years.^45^ Overall, observational studies in LMICs provide evidence that serum or urinary markers of choline concentration are positively associated with child growth, although stronger study designs must test this connection.

#### Choline during the first 1000 days

To our knowledge, no trials or observational studies in LMICs have reported on the association between choline intake or plasma choline concentration spanning pre- and postnatal periods and child neurodevelopment, growth, morbidity, or inflammation.

#### Limitations and future directions

More information is needed regarding the role of perinatal choline in LMICs on all child health outcomes. Both of the reviewed RCTs enrolled alcohol consumers, and neither assessed dose-response relationships or stratified by baseline choline intake, limiting the ability to refine choline intake recommendations. Given that choline’s influence on neurodevelopment is hypothesized to extend from pregnancy through complementary feeding, potentially up to year 4,^25^ studies of choline supplementation across this period are needed. Studies should also examine the effects of prenatal choline on birth size, because some studies suggest smaller length or weight after prenatal supplementation.

### Docosahexaenoic acid

#### Proposed mechanisms

DHA is a long-chain polyunsaturated fatty acid (LC-PUFA) highly concentrated in brain and retinal tissues, where it influences neural and visual development. In animal models, perinatal supplementation with DHA improved performance on cognitive tests,^23^ and prenatal deficiency was associated with poorer cognitive performance.^46^ Comprehensive reviews of DHA’s mechanisms may be found elsewhere.^22^ Briefly, increased DHA levels promote neural development, including formation of hippocampal synapses.^47^ Phospholipid-bound DHA in retinal membranes influences visual signaling pathways by interacting with rhodopsin.^48^ DHA is also a ligand for cell surface receptors such as GPR120, influencing anti-inflammatory cell-signaling pathways,^49^ and transcription factors, influencing gene expression in the brain.^50^^,^^51^ DHA is a precursor for a myriad of anti-inflammatory metabolites, including resolvins and neuroprotectins^52^; in producing these metabolites, DHA blocks metabolism of ARA to pro-inflammatory eicosanoids, including prostaglandins and leukotrienes. Because these metabolites have important physiological functions, balance of DHA and ARA during early life seems necessary for optimal development.^53^

DHA is well known for its anti-inflammatory actions, including creation of anti-inflammatory eicosanoids, decreased production of inflammatory cytokines, and altered cell signaling.^54^ These changes affect development of immune function in infants, as well.^55^ Prenatal DHA supplementation is associated with a more mature infant immune system (characterized by improved oral tolerance and a more balanced T-helper cell 1 and T-helper cell 2 response) in humans.^56^

LC-PUFAs including DHA are associated with longer gestation and larger birth weight,^57^ perhaps due to altered production of eicosanoids involved in parturition.^58^ DHA may also promote prenatal growth via changes in gene expression. Changes in methylation of genes related to fetal growth and development (*IGF2*/*H129*) were reported after prenatal DHA supplementation, only among preterm infants or overweight mothers.^59^ It is unclear if these changes in methylation could affect postnatal growth.

#### DHA during pregnancy

##### Designs of reviewed studies

Seven trials examined the effects of prenatal DHA supplementation in LMICs. Neurodevelopment and pregnancy outcomes were the primary focus of the trials identified. In Mexico, the Prenatal DHA (Omega-3 Fatty Acid) Supplements on Infant Growth and Development (POSGRAD) trial randomly assigned 1094 women to 400 mg/d algal DHA vs placebo during the second half of pregnancy.^60^ The authors of this study reported a range of outcomes, and the study was unique in supplementing DHA alone, without other LC-PUFAs. In Bangladesh, 400 women were randomly assigned to consuming fish oil daily (containing 1.2 g DHA, the largest dose among reviewed trials) vs soy oil capsules daily throughout the third trimester, and neurodevelopmental, growth, and birth outcomes were reported.^61^ Five RCTs reported only pregnancy outcomes, including a trial in China,^62^ 3 trials in Iran,^63–65^ and 1 in Egypt.^66^ Of note, the trial by Ostadrahimi et al^63^ is included in this discussion of pregnancy outcomes because participating pregnant women were provided prenatal supplementation; however, supplementation continued after birth, and further discussion of this trial is included later in the section on pre- and postnatal DHA. This was also the only trial to report DHA status at baseline.

Seven observational studies were identified, including 2 in Mexico (1 reporting only neurodevelopmental outcomes^67^ and 1 reporting pregnancy outcomes, child growth, and inflammation^68^) and 5 in India, which only reported birth outcomes.^69–73^

##### Neurodevelopment and visual development

In Bangladesh, there was no improvement in infant BSID-II scores at 10 months after maternal supplementation with a large dose of fish oil.^61^ Similarly, the POSGRAD study in Mexico reported no group differences in brainstem auditory-evoked potentials at 1–3 months, visual-evoked potentials at 3–6 months,^74^ BSID-II scores at 18 months,^75^ or McCarthy Scales of Children’s Abilities scores at 5 years.^76^ However, compared with control children, prenatal DHA supplementation was associated with improved sustained attention at 5 years as measured by the percentage of children scoring < 40 on the omissions subtest of the Conners’ Kiddie Continuous Performance Test (14.4% vs 25.7%; *P* < 0.0001).^76^ No association was found between DHA intake during the third trimester and brainstem auditory-evoked potentials at 1–3 months in an observational study of 76 Mexican women.^67^

The scant evidence suggests little effect of prenatal DHA on neurodevelopment or visual processing in LMICs. Studies with prolonged follow-up are needed to determine if the delayed benefit to attention reported in the POSGRAD trial is consistent across other studies.

##### Pregnancy outcomes

Of the 7 RCTs in which the effects of prenatal DHA supplementation on birth outcomes were examined, 3 trials reported significant effects (Table 2^60^^,^^61–66^^,^^68–73^). In Iran, healthy pregnant women receiving fish oil had fewer LBW infants compared with a control group (0% vs 6.7%; *P* = 0.02)^63^; however, similar studies among Iranian women with gestational diabetes reported null effects.^64^^,^^65^ In the Mexican POSGRAD study, there were no differences in birth outcomes between groups except after stratification by gravidity. Among primigravid mothers, prenatal DHA supplementation was associated with heavier babies with larger head circumference and lower risk of LBW and intrauterine growth restriction.^60^ In Egypt, women with asymmetrical intrauterine growth restriction pregnancy, as measured via ultrasound, were given aspirin with or without omega-3 fatty acids for 6 weeks during the third trimester. The omega-3 group had greater estimated fetal weight gain during the intervention and larger birth weight at delivery, compared with those who received aspirin alone.^66^

**Table 2:** Studies describing the role of prenatal DHA on pregnancy outcomes in low- and middle-income countries

Reference		Participants	Type of exposure	Timing of exposure measurement		Pregnancy outcomes	Results
Randomized controlled trials							
Ali et al (2017)^66^		*n* = 80 pregnant women in Egypt with asymmetric IUGR	Omega-3 capsules (1 g fish oil) + aspirin (*n* = 40) vs aspirin alone (*n* = 40)	From gestational wk 28–30, for 6 wk		Gestational age; birth weight	Increased birth weight in the group receiving omega-3 with aspirin (2022, SD 25 vs 2324, SD 19 g, *P* < 0.01).
Jamilian et al (2016)^64^		*n* = 56 pregnant women in Iran with gestational diabetes	Omega-3 pearls (120 mg DHA, 180 mg EPA) (*n* = 27) vs placebo (*n* = 27)	From gestational wk 24–28, for 6 wk		Gestational age; birth weight, length, head circumference	No significant differences between groups.
Olsen et al (2019)^62^		*n* = 5118 pregnant women in China	2 g fish oil (*n* = 1706) vs 0.5 g fish oil (*n* = 1695) vs placebo (*n* = 1717)	Gestational wk 16–24 until gestational week 37		Gestational age; preterm birth, early preterm birth, early term birth	No significant differences between groups.
Ostadrahimi et al (2017)^63^		*n* = 150 pregnant women in Iran	Fish oil (120 mg DHA, 180 mg EPA, 400 mg ALA; *n* = 75) vs liquid-paraffin placebo (*n* = 75)	Gestational week 20 until delivery		Gestational age; preterm birth; birth weight, length, and head circumference; LBW	Fewer LBW infants in the fish oil group than in the placebo group (0% vs 6.7%; *P* = 0.02).
Ramakrishnan et al (2010)^60^		*n* = 1094 pregnant women in Mexico	400 mg algal DHA capsules (*n* = 547) vs placebo (*n* = 547)	Gestational wk 18–22 until delivery		Gestational age; preterm birth; birth weight, length, and head circumference; IUGR	Among primigravid mothers, birth weight was 99.4 g (95%CI, 5.5–193.4g) heavier, head circumference was 0.5 cm (95%CI, 0.1–0.9 cm) larger, and risk of LBW and IUGR were lower in the DHA group.
Razavi et al (2017)^65^	*n* = 120 pregnant women in Iran with gestational diabetes		Randomized 1:1:1:1 to omega-3 capsules (240 mg DHA, 360 mg EPA), 50 000 IU vitamin D, both, or control	From gestational wk 24–28, for 6 wk	Gestational age; preterm birth; birth weight, length, head and circumference		No significant differences between groups.
Tofail et al (2006)^61^		*n* = 400 pregnant women in Bangladesh	4 g fish oil (1.2 g DHA, 1.8 g EPA) (*n* = 200) vs soy oil placebo (*n* = 200)	Gestational week 25 until delivery	Gestational age; preterm birth; birth weight, length, head circumference		No significant differences between groups.
Observational studies							
Al-Hinai et al (2018)^68^		*n* = 236 pregnant women in Mexico	Intake of fatty acids	Median 13.0 wk of gestation; 24.7 wk of gestation; 37.0 wk of gestation		Birth weight and length; gestational age	Second-trimester DHA intake was negatively associated with birth weight (−0.07 kg per SD [95%CI, −0.12, −0.02]) and length (−0.34 cm [95%CI, −0.59, −0.09]).
Dhobale et al (2011)^72^		*n* = 102 pregnant women in India, categorized into preterm or term	Placental fatty acids	At delivery		Preterm vs term; birth weight, length, head and chest circumference	Placental DHA level was lower in the preterm group than in the term group (2.05 , SD 0.97 g/100 g fatty acids vs 3.19, SD 0.94 g/100 g fatty acids; *P* < 0.01).
Kilari et al (2011)^73^		*n* = 235 pregnant women in India, categorized into LBW or NBW	Maternal and umbilical plasma and RBC fatty acids	At delivery		LBW vs NBW	Higher cord plasma DHA levels in LBW group (*P* = 0.022). Among female infants, lower plasma and RBC DHA levels in the LBW group (*P* = 0.031).
Meher et al (2016)^70^		*n* = 111 pregnant women in India, categorized into LBW or NBW	Maternal and umbilical plasma and RBC fatty acids	16–20 wk of gestation; 26–30 wk of gestation; At delivery		LBW vs NBW; birth weight, length, head and chest circumference	Positive associations between maternal RBC DHA levels at 16–20 wk and birth weight (*r* = 0.222; *P* = 0.025), and maternal RBC DHA at delivery and baby head circumference (*r* = 0.241; *P* = 0.027).
Meher et al (2016)^71^		*n* = 78 pregnant women in India, categorized into LBW or NBW	Placental fatty acids	At delivery		LBW vs NBW; birth weight, length, head and chest circumference; gestational age	Placental DHA level was lower in the LBW group than in the NBW group (2.18, SD 0.56 g/100g fatty acid vs 2.53, SD 0.78 g/100g fatty acid; *P* = 0.032). Positive association between placental DHA level and birth weight (*r* = 0.325; *P* = 0.011).
Wadhwani et al (2015)^69^		*n* = 109 pregnant women in India	Maternal and umbilical plasma fatty acids	16–20 wk of gestation; 26–30 wk of gestation; At delivery		Birth weight, length, head and chest circumference	Positive association between maternal plasma omega-3 fatty acids at 16–20 wk of gestation and baby chest circumference (*r* = 0.236; *P* < 0.05).

*Abbreviations:* ALA, α-linolenic acid; DHA, docosahexaenoic acid; EPA, eicosapentaenoic acid; IUGR, intrauterine growth restriction; LBW, low birth weight; NBW, normal birth weight; RBC, red blood cell.

Of the 6 observational reports, 5 were from prospective studies in India that followed women through pregnancy and delivery. Two reports noted positive associations between maternal plasma or RBC DHA levels and birth size.^69^^,^^70^ Two others reported lower placental DHA in preterm and LBW babies compared with term and normal weight babies,^70^^,^^72^ although in 1 report, researchers found higher cord plasma levels of DHA among LBW newborns.^73^ Interestingly, an observational study in Mexico found negative associations between second trimester dietary intake of DHA, EPA, and ARA with birth weight and length.^68^ The authors suggest this may have been due to concomitant intake of toxins like mercury or substitution of fish in place of other animal source foods, rather than a negative effect of DHA itself.^68^

Overall, evidence suggests a positive effect of prenatal DHA on birth outcomes, especially birth weight, in LMICs. Several trials had relatively small sample sizes (*n* = 4 studies with *n* ≤ 150 participants), perhaps limiting the ability to detect differences in preterm birth or gestational age, although the 2 largest trials (*n* > 1000 participants)^60^^,^^62^ also reported null effects on these outcomes. More research is needed to understand the context in which DHA, with or without other nutrients in fish oil, may affect birth outcomes. Effects may vary on the basis of maternal characteristics such as gravidity and pregnancy risk; these characteristics should be recorded in future studies.

##### Child growth

Growth outcomes in LMICs were reported in 3 studies. In Bangladesh, mean weight-for-height *z* scores (WHZ), WAZ, and HAZ at age 10 months were moderately low (−0.6 to −1.3) and not different between intervention and control groups.^61^ Among primigravid mothers in the POSGRAD study, children in the DHA group were 0.7 cm longer than control children at age 18 months (95%CI, 0.1–1.3; *P* = 0.02).^77^ This effect was lost over time, with no differences in child growth between DHA and control groups at 60 months.^78^ In an observational study in Mexico, maternal intake of DHA, EPA, and ARA during the second trimester was negatively associated with child height and body mass index (BMI) *z* score at ages 8–14 years.^68^ In each of these studies, the relationship of DHA with postnatal growth closely mirrored the relationship found with birth size. Possibly, these results simply reflect altered prenatal growth. Additional studies in LMICs may help uncover relationships between DHA and postnatal growth. Considering the opposing effects on linear growth between the 2 Mexican studies, more information is needed on DHA’s effects specific to height.

##### Morbidity and inflammation

In the POSGRAD trial, Mexican infants whose mothers were supplemented with DHA had fewer cold symptoms at 1 and 3 months than did control infants (respectively: 37.6% vs 44.6%, *P* < 0.05; and 37.8% vs 44.1%, *P* < 0.05).^79^ At 3 months, the DHA group spent 14% less time sick than did the control group.^79^ Children in the DHA group also had fewer respiratory symptoms through 18 months of age, but only among children whose mothers were atopic.^80^ In the observational study in Mexico, there was no association between maternal DHA intake and children’s C-reactive protein level or other markers of metabolic risk at 8–14 years.^68^ More studies in varied contexts are needed to better understand this relationship.

#### DHA from birth to 2 years

##### Designs of reviewed studies

Eight trials provided DHA during the early postnatal period (0–2 years) in LMICs. Generally, trials provided DHA either directly to breastfeeding infants or via inclusion in infant formula or total parenteral nutrition (TPN). Currently, DHA is recommended for inclusion in infant formulas^81^; however, inclusion is not required and may not occur in some LMICs.^82^ Many trials focused on neurodevelopment or visual development (Table 3^83^^,^^84–96^), including 3 from Turkey,^84^^,^^87^^,^^88^ and 1 each from Taiwan,^86^ The Gambia,^89^ Ethiopia,^83^ and Egypt.^85^ Of these trials, 2 provided fish oil directly,^83^^,^^89^ 2 provided fish oil via TPN,^84^^,^^87^ and 3 supplemented infant formula with DHA alone^88^ or DHA with ARA.^85^^,^^86^ Additionally, a trial in Malawi reported on gut permeability and growth after supplementation with a micronutrient powder and fish oil.^97^ Half of the trials^83^^,^^85^^,^^89^^,^^97^ reported DHA status at baseline.

**Table 3:** Studies describing the role of postnatal DHA level on neurodevelopment and visual development in low- and middle-income countries

Reference	Participants	Type of exposure	Timing of exposure	Developmental measure(s)	Child’s age at measurement	Results
Randomized controlled trials						
Argaw et al (2019)^83^	*n* = 360 mother-child dyads in Ethiopia	Randomized 1:1:1:1 to maternal intervention (fish oil [215 mg DHA, 285 mg EPA]), infant intervention (169 mg DHA, 331 mg EPA), both, or control	Starting at 6–12 mo old, for 1 y	Culturally adapted Denver II Developmental Screening Test (Denver II- Jimma); Ages and Stages Questionnaire: Social Emotional domain	18–24 mo old	No significant difference across groups.
Beken et al (2014)^84^	*n* = 80 VLBW preterm infants (< 32 wk gestation) in Turkey	SMOFlipid^a^ (*n* = 40) vs standard lipid emulsion (*n* = 40)	Birth until weaning from TPN (mean age, 14 d)	Retinopathy of prematurity diagnosis; need for laser photocoagulation of the retina	Birth until hospital discharge (mean age, 34 d)	Control group had higher odds of retinopathy of prematurity than the group receiving SMOFlipid (OR, 9.1; 95%CI, 1.9–43.8). No difference between groups in need for laser photocoagulation.
El-khayat et al (2007)^85^	*n* = 42 term infants in Egypt with WHZ < −2	Infant formula supplemented with 0.01 g/100 mL DHA, 0.02 g/100 mL ARA (*n* = 21) vs standard formula (*n* = 21)	Starting at 6–25 mo old, for 8 wk	BSID II (MDI and PDI scores)	Baseline (6–25 mo old); Endline (8–27 mo old)	Larger mean change in MDI and PDI scores in the supplementation group than in control group. Positive correlations between plasma DHA level and MDI (*r* = 0.52) and PDI (*r* = 0.50; *P* < 0.05 for both).
Fang et al (2005)^86^	*n* = 27 preterm infants (30–37 wk of gestational age) in Taiwan	Infant formula supplemented with 0.05% DHA, 0.10% ARA (*n* = 16) vs standard formula (*n* = 11)	Birth until 6 mo old	Visual acuity: visual evoked potentials, Lea grating acuity cards, Hiding Heidi low-contrast “FACE” cards;BSID (MDI and PDI scores)	4 mo old; 6 mo old; 1 yr old;	No significant differences in visual acuity measures between groups. There was a significant difference in MDI and PDI scores between groups via repeated measures ANOVA, with higher scores in the supplemented group.
Ozkan et al (2019)^87^	*n* = 89 preterm infants (<32 wk of gestation) in Turkey	SMOFlipid^a^ (*n* = 42) vs standard lipid emulsion (*n* = 47)	Birth until weaning from TPN (mean age, 13 d)	Retinopathy of prematurity diagnosis	Birth until hospital discharge (mean age not provided)	No significant difference between groups.
Unay et al (2004)^88^	*n* = 54 term newborns in Turkey who received formula, and *n* = 26 breastfeeding control infants	Infant formula supplemented with 0.5g DHA/100g lipids (*n* = 28) vs standard formula (*n* = 26) vs breast milk (*n* = 26)	Birth until 16 wk old	Brainstem auditory evoked potentials (absolute wave and interpeak latencies describe response to auditory stimuli)	1 wk old; 16 wk old	All latencies decreased from birth to 16 wk; the group receiving standard formula had smaller decreases than the DHA-supplemented or breastfed groups (*P* < 0.05 for all).
van der Merwe et al (2013)^89^	*n* = 183 infants in The Gambia	Fish oil containing 200 mg DHA, 300 mg EPA (*n* = 92) vs olive oil placebo (*n* = 91)	Starting at 3 mo old, for 6 mo	Willatt’s Infant Planning Test; Toddler attention assessment	1 yr old	No significant difference between groups.
Observational studies						
Fahmida et al (2015)^90^	*n* = 240 children of Sasak ethnicity in Indonesia, categorized by genotype	*FADS* genotype (involved in LC-PUFA production); Child plasma fatty acids	12–17 mo old	BSID II (MDI score)	12–17 mo old	Genotype was not significantly associated with MDI score; however, the log DHA-to-EPA ratio was associated with MDI score (β = 1.75; 95%CI, 0.08–3.41).
Gharehbaghi et al (2020)^91^	*n* = 341 preterm infants (<2000 g, <34 wk of gestational age) in Iran	SMOFlipid^a^ vs standard lipid emulsion	Birth until weaning from TPN (mean age, 14 d)	Retinopathy of prematurity diagnosis	Birth until final follow-up (age not provided)	No significant difference between groups.
Henjum et al (2018)^92^	*n* = 320 infants in Nepal	Infant RBC fatty acids	2–11 mo old	Ages and Stages Questionnaire-3; NEPSY II subtests	5 y old	No significant association between RBC DHA level and neurodevelopmental scores.
Krasevec et al (2002)^93^	*n* = 56 infants in Cuba	Infant and maternal plasma and RBC fatty acids; Breast milk fatty acids	2 mo old	Visual acuity measured via Teller acuity cards	2 mo old	No significant associations between visual acuity scores and fatty acid concentrations.
Luxwolda et al (2014)^94^	*n* = 97 infants from 3 tribes in Tanzania plus 15 Dutch infant controls	Tribal fish intake level (low, intermediate, high); Infant RBC fatty acids	10–20 wk old	General movement quality measured via Assessment of Motor repertoire	10–20 wk old	Children in the high-fish-intake tribe had improved observed movement patterns compared with Dutch control children; no difference between tribes. RBC-DHA level was associated with observed movement patterns score (β = 0.304; 95%CI, 0.061, 0.547).
Marín et al (2000)^95^	*n* = 28 term, moderately underweight (WAZ between −2 and −3) infants in Argentina	LC-PUFA supplemented formula vs standard formula vs breast milk; Infant RBC fatty acids	45–90 d old	Full-field flash electroretinography (the b-wave latency describes retinal response to light stimuli)	45–90 d old	Standard formula group had longer b-wave latencies (mean ± SD: 73.8 ± 7.4 ms) compared with LC-PUFA or breast milk groups (52.0 ± 5.4 and 51.3 ± 1.0). Correlation between infant RBC DHA and b-wave latency (*r*^2^ = 0.96; *P* < 0.0001).
Unal et al (2018)^96^	*n* = 227 VLBW preterm infants (25–32 wk of gestational age) in Turkey	SMOFlipid^a^ vs standard lipid emulsion	Birth until weaning from TPN (mean age, 7 d)	Retinopathy of prematurity diagnosis	Birth until hospital discharge (mean age, 45 d [fish oil group] and 48 d [control group]; *P* = 0.317)	No significant difference between groups.

*Abbreviations:* ANOVA, analysis of variance;ARA, arachidonic acid; BSID, Bayley Scales of Infant Development; DHA, docosahexaenoic acid; EPA, eicosapentaenoic acid; LC-PUFA, long-chain polyunsaturated fatty acid; MDI, Mental Developmental Index; OR, odds ratio; PDI, Psychomotor Developmental Index; TPN, total parenteral nutrition; VLBW, very low birth weight; WAZ, weight-for-age *z* score; WHZ, weight-for-height *z* score.

a In contrast to standard emulsions, SMOFlipid (Fresenius Kabi) adds fish oil, medium-chain triglycerides, and higher levels of α-tocopherol.

Ten observational studies described DHA in plasma, RBCs, lipid emulsion or breast milk, and neurodevelopment or growth outcomes across a range of LMICs.

##### Neurodevelopment and visual development

Of 7 studies in which visual development was measured, only 2 reported a significant relationship between DHA and visual development (Table 3). In a trial in Turkey, researchers reported that addition of fish oil to TPN emulsions reduced risk for retinopathy of prematurity among very-low-birth-weight preterm infants.^84^ However, null results were reported in 3 similar studies in Turkey and Iran of preterm infants receiving TPN.^87^^,^^91^^,^^96^ In Argentina, malnourished infants who consumed standard formula had poorer retinal response to light stimuli compared with those who consumed LC-PUFA–supplemented formula or breast milk^95^; however, the study was small (*n* = 28), observational, and researchers did not correct for potentially confounding factors, such as socioeconomic status or maternal education. The 1 study involving healthy, term children in Cuba found null associations between plasma, RBC, or breast milk DHA concentrations and visual development.^93^

Of 8 studies reporting neurodevelopmental outcomes, 3 RCTs and 2 observational studies reported significant results (Table 3). Among RCTs, the 3 that supplemented infant formula with DHA, with or without other fatty acids, reported significant improvements in neurodevelopment.^85^^,^^86^^,^^88^ In both RCTs which directly supplemented breastfeeding infants or lactating women, null results were reported.^83^^,^^89^

An observational study in Indonesia found that although genotype of the *FADS* gene cluster, involved in endogenous production of LC-PUFAs, was not related to the BSID-II Mental Development Index at ages 12–17 months, the plasma DHA-to-EPA ratio was positively associated with this score.^90^ In Tanzania, RBC DHA was positively associated with movement patterns at 10–20 weeks of age.^94^ However, there was no association between RBC DHA in infancy and neurodevelopment at 5 years in children in Nepal.^92^

Overall, there is little evidence that DHA supplementation improves visual or neural development for healthy, breastfeeding children in LMICs. However, benefits to visual development were seen among malnourished or hospitalized infants, and there is supportive evidence for including DHA in infant formula. There may be a relationship between plasma or RBC DHA and neurodevelopment, limited to specific populations or developmental domains.

##### Child growth

Seven studies in LMICs included measures of child growth. Among trials, changes in body composition and adiposity were commonly noted. In The Gambia, infants who received fish oil had larger mid-upper arm circumference for their age and triceps skinfold thickness for age compared with control infants.^89^ In Ethiopia, fish oil provision to breastfeeding infants, but not to lactating women, was associated with increased monthly WHZ gains compared with control infants.^98^ In a Malawian trial, children aged 12–35 months who received micronutrient powder with fish oil gained more weight over 24 weeks than did control children (1.3 kg vs 1.1 kg; *P* = 0.01).^97^ There was no difference in linear growth, and no other anthropometrics were reported. In Taiwan, in a trial of preterm infants, researchers found no differences in child height, weight, or head circumference with DHA-supplemented vs traditional formula; no measures of body composition were reported.^86^

Authors of 3 observational studies have reported a relationship between DHA in serum or breast milk and child height and weight; none included other anthropometric indices. In Malawi, serum DHA and ARA concentrations were positively associated with HAZ among 400 Malawian children aged 12–59 months.^99^ In a small sample in China (*n* = 41), breast milk DHA was positively related to postnatal length gain at 1 month (*r* = 0.83) and 3 months (*r* = 0.76; *P* < 0.01 for both) and weight gain at 3 months (*r* = 0.46; *P* < 0.05).^100^ In the Congo and Burkina Faso, children’s monthly weight gain from birth to 5 months was examined in association with breast milk fatty acid content. Monthly weight gain decreased as the ratio of omega-6 fatty acids to omega-3 fatty acids increased until a cutoff of 15:1, at which point weight gain remained at a steady low.^101^ Although not specific to DHA, it suggests that a substantial intake of omega 3 fatty acids is needed among lactating women with high intake of omega-6 rich oils to optimize child weight gain.

Although authors of observational studies in LMICs have noted links between DHA and child length and weight, in RCTs, effects on body composition and adiposity are more commonly reported. Few trials included supplementation of preterm infants or lactating women. Studies should investigate these populations and include a variety of anthropometric measures.

##### Morbidity and inflammation

Five studies reported on morbidity or inflammation related to postnatal DHA in LMICs. In Ethiopia, prevalence of inflammation (based on elevated C-reactive protein levels) and morbidity was not different between groups after supplementing lactating women or infants with fish oil vs a control; the authors suggested this finding may have been due to the low prevalence of inflammation and morbidity in this study compared with others.^98^ In Malawi, all participants had high ratios (> 0.1) of lactulose to mannitol at baseline, reflecting increased gut permeability, and there was no difference among children who received micronutrient powder with or without fish oil for 24 weeks compared with the control group.^97^ Similarly, in The Gambia, the average ratio of lactulose to mannitol was 0.22, and nearly half of children had elevated C-reactive protein levels. Children who received fish oil had no differences in lactulose-to-mannitol ratio, inflammatory markers, or morbidity, compared with control children.^89^

In Turkey, there were no changes in pro- or anti-inflammatory cytokine levels among preterm children randomly assigned to receive fish oil vs standard lipids in TPN. However, there was a lower prevalence of bronchopulmonary dysplasia in the fish oil group, and total antioxidant capacity was higher after 7 days, but not 14 days, of treatment.^87^ In a similar study in Turkey, provision of fish oil did not reduce morbidity or mortality rate, and total antioxidant capacity was higher in the fish oil group than in the control group, but this difference disappeared after treatment ended.^96^ Overall, there is little support for an effect of postnatal DHA on inflammation, gut permeability, or morbidity in LMICs.

#### DHA across the first 1000 days

To our knowledge, only 1 study has described pre- and postnatal DHA provision in an LMIC. In Iran, 150 women were randomly assigned to receive fish oil or liquid-paraffin placebo from 20 weeks’ gestation to 30 days postpartum.^102^ Pregnancy outcomes are reported in Table 3. Developmentally, there were no differences across the 5 domains of the Ages and Stages Questionnaire at 4 or 6 months, except higher communication scores in the fish oil group at 4 months.^102^ No differences in infant length, weight, or head circumference were noted between groups from birth to 6 months.^102^ Morbidity and inflammation data were not reported.

#### Limitations and future directions

The literature from LMICs suggests positive effects of prenatal DHA supplementation on birth weight and morbidity, with a potential delayed benefit to attention at age 5 years. Additionally, studies in LMICs support the addition of DHA to infant formula for improved neurodevelopment. Across life stages, conclusions have been limited by variations in dose, timing, vehicle, context, and co-interventions. The effects of DHA may vary with baseline DHA status; however, few trials reported this information. Many trials also lacked endline measures of status, relying on maternal report or pill counts for adherence data. Future trials should explore pre- and postnatal supplementation, including to preterm infants or lactating women.

### Choline and DHA

#### Proposed mechanisms

Beyond the individual effects of choline and DHA, the 2 may work together to improve neurodevelopment.^19^ Among malnourished pigs, addition of dietary DHA, methyl donor nutrients including choline, or both attenuated losses in fetal brain weight compared with controls.^103^ Combined choline and DHA administration decreases brain inflammation^104^ and oxidative stress^105^ in mouse models. In fact, these nutrients may work synergistically: offspring of dams supplemented with choline and DHA had more hippocampal neurons than those given either nutrient alone.^106^

This synergy reflects the interconnected nature of choline and DHA metabolism. Phosphatidylcholine incorporates DHA via the phosphatidylethanolamine *N*-methyltransferase pathway and is the main carrier of DHA in plasma, including among preterm infants.^107^ Lysophosphatidylcholine-DHA is the main form of DHA transported into the brain and eye, via the Mfsd2a transporter.^108^ Maintenance of phosphatidylcholine-DHA levels is important for neural progenitor cell proliferation.^109^ Additionally, choline and DHA affect each other’s transport and metabolism. Prenatal choline supplementation increases placental transcript abundance of DHA transporters in mice.^32^ Likewise, DHA increases choline uptake in retinal cells^110^ and stimulates production of acetylcholine in cultured cholinergic cells.^111^ When choline and DHA are provided together, circulating levels of each nutrient increase more than when provided separately.^112^^,^^113^ Although this synergy has been linked to improved neurodevelopment, its relationship with birth outcomes, growth, morbidity, and inflammation is unclear.

#### Choline and DHA during pregnancy

No studies specific to prenatal choline and DHA were identified in LMICs. Several studies of fish or egg intake were identified and included in this review, because fish and eggs are sources of choline and DHA. Although typically studied for its omega-3 fatty acids, fish also contains choline. (Fish oil, on the other hand, does not.) Eggs also provide these nutrients, although the DHA content varies. Both provide other food components as well, including neuroprotective factors like iodine or iron, and toxins like mercury. To understand the unique effects of choline and DHA, studies specific to these nutrients are needed.

Several observational studies have reported a link between maternal fish or egg consumption and birth outcomes in LMICs. Prospective cohort studies in Iran,^114^^,^^115^ Turkey,^116^ and India^117^ linked increased fish intake during pregnancy to decreased odds of LBW, although in 1 study in India, the opposite relationship was reported.^118^ Risk for preterm birth was also inversely related to fish consumption in Iran^114^^,^^115^ and Pakistan.^119^ Maternal consumption of eggs was positively associated with birth weight in Iran and India.^115^^,^^118^ We found no studies that reported on neurodevelopment, child growth, morbidity, or inflammation.

#### Choline and DHA from birth to 2 years

##### Designs of reviewed studies

Although no studies in LMICs have examined postnatal choline and DHA directly, 9 studies investigated foods containing choline and DHA along with other nutrients. In 3 RCTs, supplements fortified with choline, DHA, and other nutrients were compared with traditional supplemental foods and nonsupplement controls in Guinea-Bissau, South Africa, and Cambodia.^120–122^ In 2 RCTs, researchers examined the provision of 1 egg/day during the early complementary feeding period (6–15 months) vs a nonintervention control in Ecuador (The Lulun Project) and Malawi (The Mazira Project).^123^^,^^124^ One study in China compared the effects of nutrition education, including recommendations to provide daily egg yolks as an infant’s first food vs a nonintervention control on children’s growth.^125^ Only 1 trial presented baseline measures of choline and DHA status, in a separate article.^126^ An observational study in Haiti examined neurodevelopment,^127^ and 2 in India and Zambia studied growth.^128^^,^^129^

##### Neurodevelopment

In Guinea-Bissau, children younger than 4 years had improved working memory and better cerebral blood flow with consumption of a supplement containing DHA, choline, and other nutrients, compared with a traditional meal, but there was no difference compared with a common fortified food (Corn Soy Blend++).^120^ No other domains of development were measured. In the South African trial, a fortified, small-quantity, lipid-based nutrient supplement (SQ-LNS-plus) was associated with improved locomotor development, as measured by the Kilifi Developmental Inventory at 12 months, compared with a nonintervention control.^121^ The standard SQ-LNS was not different from nonintervention control, suggesting the additional nutrients were responsible for these findings. In the Mazira Project in Malawi, daily egg consumption did not affect children’s memory, attention, language, or personal social scores, but there were fewer children with delayed fine-motor development compared with control children (prevalence ratio, 0.59; 95%CI, 0.38–0.91).^130^ Children’s egg intake was also associated with motor, but not language, development in an observational study of 583 infants in Haiti; other developmental domains were not measured.^127^

Together, the limited evidence from LMICs suggests a benefit to neurodevelopment, especially motor development, from postnatal intake of choline- and DHA-containing foods. No studies provided these nutrients to lactating women; this may be an area for future research.

##### Child growth

Eight studies reported on child growth in LMICs (Table 4^120–126^^,^^128^^,^^129^^,^^131^). In Guinea-Bissau, the fortified supplement was associated with decreased WAZ, BMI for age, fat tissue accretion, and increased lean tissue accretion compared with the corn-soy blend among children younger than 4 years.^120^ In Cambodia, consumption of a novel ready-to-use supplemental food with choline, DHA, and other nutrients was associated with increased mid-upper arm circumference compared with that of nonintervention control children, but there were no differences in HAZ, WAZ, or weight-for-length z score.^122^

**Table 4:** Studies describing the role of postnatal foods containing choline and DHA on child growth in low- and middle-income countriesa

Reference	Participants	Type of exposure	Timing of exposure	Growth measure(s)	Timing of measurement	Results
Randomized controlled trials						
Borg et al (2020)^122^	*n* = 485 children from 28 clusters in Cambodia	Fish-based RUSF (*n* = 128) vs CSB++ (*n* = 123) vs micronutrient powder (*n* = 107) vs control (*n* = 127)	Starting at 6–11 mo old, for 6 mo	HAZ, WAZ, WHZ; MUAC	Baseline (6–11 mo of age); Endline (12–17 mo of age)	The fish-based RUSF group had higher MUAC (0.04 cm; 95%CI, 0.01–0.06) than control group but was not different from the CSB++ or micronutrient powder groups.
Guldan et al (2000)^125^	*n* = 495 children from 4 townships^ b^	Nutrition education including recommendation of egg yolks for infants (*n* = 250) vs control (*n* = 245)	1 y intervention aimed at pregnant women and infants <1 y old	HAZ, WAZ	Endline (measured infants ages 4–12 mo only)	HAZ (−1.32 vs −1.96; *P* = 0.022) and WAZ (−1.17 vs −1.93; *P* = 0.004) were higher in the nutrition education townships than in controls, only among 12-mo-old children.
Iannotti et al (2017)^123^	*n* = 163 infants in Ecuador	One egg per day (143.6 mg choline, 30 mg DHA^ c^; *n* = 83) vs control (*n* = 80)	Starting at 6–9 mo old, for 6 mo	HAZ, WAZ, WHZ, BMI *z* score; stunting and underweight	Baseline (6–9 mo old); Endline (12–15 mo old)	The egg group had increased HAZ, WAZ, WHZ, and BMI *z* score than the control group. Lower prevalence of stunting and underweight in the egg group than in the control group.
Roberts et al (2020)^120^	*n* = 1059 children in Guinea-Bissau	“NEWSUP”^ d^ (22.1 mg choline, 534 mg omega-3 fatty acids; *n* = 368) vs CSB++ (*n* = 350) vs traditional rice meal (*n* = 341)	Starting at 15 mo to 7 y old, for 23 wk	HAZ, WAZ, BMI *z* score; MUAC; lean tissue area; fat tissue area	Baseline (15 mo–7 y old); Endline (~20 mo−7.5 y old)	Among children < 4 y old, the group receiving NEWSUP had decreased WAZ, BMI for age, fat tissue area, and increased lean tissue area compared with the corn-soy blend group. Compared with the control, WAZ and MUAC were decreased.
Smuts et al (2019)^121^	*n* = 750 infants in South Africa	SQ-LNS–plus (7.8 mg choline, 75 mg DHA; *n* = 250) vs SQ-LNS (*n* = 250) vs control (*n* = 250)	Starting at 6 mo old, for 6 mo	HAZ, WAZ, WHZ; MUAC; head circumference	8 mo old; 10 mo old; 12 mo old;	Compared with control group, the SQ-LNS–plus group had higher HAZ at 8 mo (effect size: 0.11, 95% CI, 0.01–0.22) and 10 mo (0.16; 95%CI, 0.04–0.27), but not 12 mo (0.09; −0.02, 0.21).
Stewart et al (2019)^124^	*n* = 660 infants in Malawi	One egg per day (126 mg choline, 40 mg DHA,^ c^*n* = 331) vs control (*n* = 329)	Starting at 6–9 mo old, for 6 mo	HAZ, WAZ, WHZ, HCAZ; stunting, underweight, wasted, small head size	Baseline (6–9 mo old); Endline (12–15 mo old)	No difference in growth between groups except improved HCAZ (adjusted mean difference: 0.12; 95%CI, 0.49–1.42) and lower prevalence of small head size in the egg group compared with the control group.
Observational studies						
Aguayo et al (2016)^129^	*n* = 2561 children in India	Feeding practices, including consumption of eggs	0–23 mo old	HAZ; stunting status	0–23 mo old	Children ages 6–23 mo who did not consume eggs had increased odds of stunting after adjustment (OR, 2.073; 95%CI, 1.191–3.606).
Marinda et al (2018)^128^	*n* = 714 children in Zambia	Feeding practices, including consumption of fish	6–59 mo old	HAZ, WAZ, WHZ	6–59 mo old	Among children ages 6–23 mo, there was a positive correlation between fish consumption and HAZ (*r* = 0.139; *P* = 0.008).

*Abbreviations:* CSB, corn-soy blend; DHA, docosahexaenoic acid; HAZ, height-for-age *z* score; HCAZ, head circumference-for-age *z* score; MUAC, mid-upper arm circumference; OR, odds ratio; RUSF, ready-to-use supplementary food; SQ-LNS, small-quantity lipid-based nutrient supplements; WAZ, weight-for-age *z* score; WHZ, weight-for-height *z* score.

a Townships were not randomly selected.

b Nutrient values were presented in separate manuscripts for the Lulun^126^ and Mazira^131^ Projects.

c NEWSUP was a novel food supplement fortified with choline, DHA, and other nutrients, including polyphenols, chromium, and molybdenum.

Researchers have noted an effect on linear growth in several trials. In South Africa, HAZ was higher in the SQ-LNS-plus group than in the control group at ages 8 and 10 months, but not 12 months; the standard SQ-LNS group was not different from the control group.^121^ Large increases in HAZ (effect size: 0.61; 95%CI, 0.45–0.77) and WAZ (0.61; 95%CI, 0.37–0.77), as well as increases in WHZ and BMI for age, were noted after egg provision in Ecuador.^123^ However, these effects were absent 2 years later, suggesting a longer intervention may be needed to sustain benefits.^132^ In Malawi, despite a similar study design, no effects on HAZ, WAZ, or WHZ were reported after egg provision, although head circumference for age was larger in the intervention group.^124^ This difference in response may be due to the high rates of fish consumption in Malawi^124^; perhaps eggs improve growth only in the absence of choline- and DHA-containing foods in the usual diet. Baseline stunting rates were also lower in the Malawi study (14%) compared with the study in Ecuador (38%).^123^^,^^124^ In China, 12-month-old children in townships where eggs were recommended for child feeding had larger WAZ and HAZ but not WHZ, compared with children in control townships.^125^ However, these townships were not randomly selected and received additional messages about other health practices, such as breastfeeding. In India and Zambia, nonconsumption of eggs and fish by children aged 6–23 months was associated with increased risk of stunting.^128^^,^^129^ Overall, these studies suggested a beneficial effect of foods containing choline and DHA on child growth in LMICs, albeit perhaps limited to certain contexts.

##### Morbidity and inflammation

In South Africa, the SQ-LNS-plus group had decreased longitudinal prevalence of fever, coughing, and wheezing, and increased longitudinal prevalence of diarrhea, vomiting, and rashes and sores compared with the control group. These effects were not specific to choline and DHA, because the standard SQ-LNS group had similar results.^121^ In the Lulun Project in Ecuador, prevalence of diarrhea in the past 7 days was higher in the egg group than in the control group; however, the data were from parental reports, which the authors speculate may have been biased.^123^ The Mazira Project has not yet reported child morbidity outcomes. No trial has reported on inflammation.

#### Choline and DHA across the first 1000 days

No trials in LMICs have reported on pre- and postnatal relationships between choline, DHA, and child neurodevelopment, growth, morbidity, or inflammation.

#### Limitations and future directions

The literature on perinatal choline and DHA in LMICs is sparse, and no studies assessed the effects of choline and DHA independently of other nutrients. When possible, the specific effects of these nutrients, independent of other dietary factors, should be assessed. Postnatal choline and DHA doses were generally below recommendations; however, improvements to neurodevelopment and growth were evident even at these levels. Given these promising findings, more trials in diverse contexts should be prioritized.

## DISCUSSION

Overall, limited data suggest improvements in child development, birth outcomes, growth, morbidity, and inflammation related to perinatal provision of choline, DHA, and a combination of the 2 nutrients in LMICs. There is evidence to suggest that supplementation with these nutrients may be beneficial for pregnant and lactating women and young children. However, more research is needed to address the following questions.

### What are the specific long-term effects of choline and DHA during early life in LMICs?

Additional studies are required to understand the effects of varying doses of choline and/or DHA on child health in LMICs. Trials should use high-quality physiological measures of child development, such as eye-movement response time and heart rate, and accurate biomarkers. Measures such as eye tracking are feasible in LMICs^133^ but may require more funding and training than assessments based on acquisition of developmental milestones. Accurate biomarkers of intake and status will be required across all settings for better measurement of exposure and understanding of biological effects. Controlled feeding trials with varying dosages in multiple arms, although challenging, would provide high-quality evidence and are lacking in LMICs. Studies with prolonged follow-up are needed to understand the long-term impacts on health and productivity.

### In what settings would choline and DHA supplementation be beneficial?

Although intake of choline and DHA is thought to be low in many LMICs, this is not the case in all settings. Coastal populations may have substantial intake of fish, regardless of income.^12^ A useful example of this concept is a comparison between the Mazira and the Lulun Projects. Both trials provided eggs to young children in LMICs, but the results on child growth were strikingly different between populations.^123^^,^^124^ The investigators suggested several possible reasons for this contrast, including differences in background fish intake (high intake near Lake Malawi; low intake in highland Ecuador).^124^ Indeed, in Malawi, breast milk DHA concentrations among women living near the lake are higher than the global average.^134^ Especially in areas with adequate intake of animal source foods, choline and DHA may not be limiting nutrients for children’s growth and development. There is a need for more information on population choline and DHA status as well as usual dietary intake. Incorporation of choline and DHA into national nutrition monitoring systems and food composition databases is needed to inform future interventions. Databases should include the 5 chemical forms of choline, which may have variable effects on children’s health,^38^ as well as betaine, a separate dietary component that may have a choline-sparing effect and is worthy of more research.

### How might choline and DHA fit into local, sustainable, and affordable diets?

Considering the perinatal benefits of choline and DHA, efforts to increase maternal and infant intake of these nutrients are needed in LMICs. Breast milk is a good source of these nutrients and should be recommended as the only food for infants up to age 6 months; however, the concentrations in breast milk vary by maternal diet,^8^^,^^9^ and complementary food sources of these nutrients are needed after 6 months. The main food sources of these nutrients are often relatively expensive, and there are concerns about sustainability and environmental issues related to their production. Alternative food products, such as biofortified foods, may be needed to meet global maternal and infant needs affordably and sustainably.

Where food sources are unavailable or inappropriate, supplementation is an option. Choline is required and DHA is recommended for inclusion in infant formula,^81^ and choline is recommended in prenatal supplements,^135^ but products meeting these recommendations may not be available or affordable in LMICs. Choline is supplemented as choline salts, such as choline bitartrate, or phosphatidylcholine. DHA is often supplemented as either fish oil or algal oil. Krill oil contains DHA linked to phospholipids including phosphatidylcholine and has similar bioavailability to fish oil; however, it is expensive and has similar sustainability constraints.^136^

## CONCLUSION

More research is needed on the role of choline and DHA during the first 1000 days on child outcomes in LMICs. Dose-response trials are necessary to refine nutrient intake requirements, and measures of population status should be incorporated into national nutrition programs. This would enable better monitoring of global dietary adequacy as well as improved formulation of fortified or supplementary foods. At this time, adequate intake of foods rich in choline and DHA should be recommended for pregnant and lactating women and their young children, including breast milk for infants.
